# Highly Damage-Resistant Thin Film Saturable Absorber Based on Mechanically Functionalized SWCNTs

**DOI:** 10.1186/s11671-021-03648-2

**Published:** 2022-01-15

**Authors:** Daewon Kang, Sourav Sarkar, Kyung-Soo Kim, Soohyun Kim

**Affiliations:** 1 Department of Mechanical Engineering, Korea Advanced Institute of Science and Technology, 34141 Daejeon, Republic of Korea; 2 K LAB Co., Ltd., 34014 Daejeon, Republic of Korea

**Keywords:** Saturable absorber, Mode-locking, All-fiber laser, Thermal damage

## Abstract

Thin-film saturable absorbers (SAs) are extensively used in mode-locked fiber laser due to the robust and simple application methods that arise because SAs are alignment-free and self-standing. Single-walled carbon nanotubes (SWCNTs) are the most suitable low dimensional material uesd for SAs because of their high nonlinearity and the wavelength control of absorption based on tube diameters. The most challenging problem with the use of CNT-based thin film SAs is thermal damage caused during high power laser operation, which mainly occurs due to aggregation of CNTs. We have demonstrated improved thermal damage resistance and enhanced durability of a film-type SA based on functionalization of SWCNTs, which were subjected to a mechanical functionalization procedure to induce covalent structural modifications on the SWCNT surface. Increased intertube distance was shown by X-ray diffraction, and partial functionalization was shown by Raman spectroscopy. This physical change had a profound effect on integration with the host polymer and resolved aggregation problems. A free-standing SA was fabricated by the drop casting method, and improved uniformity was shown by scanning electron microscopy. The SA was analyzed using various structural and thermal evaluation techniques (Raman spectroscopy, thermogravimetric analysis, etc.). Damage tests at different optical powers were also performed. To the best of our knowledge, a comprehensive analysis of a film-type SA is reported here for the first time. The partially functionalized SWCNT (fSWCNT) SA shows significant structural integrity after intense damage tests and a modulation depth of 25.3%. In passively mode-locked laser operation, a pulse width of 152 fs is obtained with a repetition rate of 77.8 MHz and a signal-to-noise ratio of  75 dB. Stable operation of the femtosecond fiber laser over 200 h verifies the enhanced durability of the fSWCNT SA.

**Supplementary Information:**

The online version contains supplementary material available at 10.1186/s11671-021-03648-2.

## Introduction

Passively mode-locked erbium-doped fiber lasers (EDFLs) have been popular in generating ultrashort optical pulses with widespread applications in industrial and scientific fields. Complex technical areas such as high-resolution microscopy, biophotonics, optical signal processing and optical metrology have extensively employed EDFLs due to their simple, reliable operation and consistent ability to operate in the picosecond and femtosecond regimes [1–5]. The working principle of a mode-locked fiber laser closely depends on the nonlinearity of an optical element called a saturable absorber (SA) present in the laser cavity whose intensity-dependent response is crucial for ultrashort pulse generation. Although the mode-locking technology based on semiconductor saturable absorber mirrors (SESAMs) is widely applied, the high cost and complexity in operation have had adverse effects on their wide-ranging application [6, 7].

Low-cost manufacturing combined with attractive features such as high nonlinearity has enabled the use of low-dimensional materials such as carbon nanotubes (CNTs) or graphene as alternatives to SESAMs. Film-type SAs with a free-standing capability can be applied to all fiber lasers in the simplest way. However, low-dimensional material-based SA films have also suffered significantly from low thermal stability and a high degradation rate owing to unreliable preparation processes, rendering them unsuitable for durable laser applications. Several studies using microfiber (for evanescent wave interactions) have been performed to overcome these limitations, but polarization sensitivity and high loss problems are caused by side-polished fibers [8, 9]. Additionally, tapered fibers with a few µm waist diameter are broken by minor impacts and exposure to environmental changes for a long time [10]. Recently, various studies have been performed to improve the damage resistance of SAs with tungsten disulfide, MoS$_{2}$ and In$_{2}$Se$_{3}$ [11–13].

Saturable absorption is a nonlinear phenomenon in which photonic materials undergo saturation of optical absorption under high-intensity illumination, which constitutes the basis of passive mode locking [14, 15]. Although all materials show a certain degree of saturable absorption, it occurs close to their optical damage threshold. Conversely, in CNTs, this absorption occurs at modest light intensities, while varying diameters offers better control over the operational wavelength [16]. Some of the key requirements of ideal SAs include superior control over saturable absorption and thermal durability to ensure stable, uninterrupted laser operation. CNT-polymer composite SAs with ease of producing large-area thin films with optical uniformity have the potential to satisfy some of these conditions [17]. One of the reasons behind the current capability limitations of SAs is the lack of uniformity in the distribution of CNTs in the polymer matrix, which greatly reduces the overall composite film performance. Conventional dispersion methods using surfactants such as sodium dodecyl sulfate (SDS) have some drawbacks (increased optical loss due to impurities, etc.). We present a solution to this problem by using fCNTs in a polymeric double layer film framework, which shows remarkable thermal stability and damage resistivity under stringent laser operation. Several reported works on high-performing composite polymers involved surface modification of graphene through ultrasonication techniques [18]. We hypothesized that mechanical modification or functionalization will be helpful in the formation of stable dispersions of CNTs in solvent media, which in turn will lead to perfect integration of nanofillers (CNTs) into the polymer matrix [19, 20]. Composite materials with uniformly dispersed nanofillers in the polymer matrix have displayed improved structural integrity combined with a higher thermal threshold in previously reported works [21].

As presented in this paper, SWCNTs were partially functionalized, resulting in an increase in the intertube distance. The improved uniformity of the the functionalized SWCNT (fSWCNT) SA is shown by scanning electron microscopy (SEM) images. Structural and chemical evaluation of SAs was also performed by applying X-ray diffraction (XRD), Raman and Fourier transform infrared (FTIR) techniques. The fSWCNT SA is proven to be thermally stable at high temperatures exceeding 650 $^{\circ }{\mathrm {C}}$ according to thermogravimetric analysis (TGA) and to exhibit sustained damage resistivity in an EDFL operating at 1550 $\text {nm}$ over a wide range of output powers. After exposing the SA to high-power continuous waves in the 1550 nm range, it was applied to an EDFL, and stable mode locking was confirmed. To our knowledge, this is the first comprehensive analysis of damage resistance involving one-dimensional material-reinforced polymer SA films. Our results demonstrate that fSWCNT SAs exhibit remarkable performance, including high damage resistance, due to the uniform distribution and femtosecond pulse generation in EDFLs. This new class of damage-resistant SAs is suitable for mass production at a low cost with good reproducibility, so it has the potential to usher in more advanced development of low-dimensional material-based polymeric SAs in the future.

## Materials

CNTs are classified as single-walled CNTs (SWCNTs) and multiwalled CNTs (MWCNTs) according to the number of layers constituting them. SWCNTs are cylinders composed of a layer of a graphene strip, and the structural characteristics can be defined in terms of the chirality by a chiral vector ($C_{\mathrm{h}}$). The width of the strip is equal to the circumference of the SWCNT and can be expressed as

1 $$\begin{aligned} C_{\mathrm{h}}=n\vec {a_1}+m\vec {a_2}\equiv (n,m) \end{aligned}$$

where $\vec {a_1}$ and $\vec {a_2}$ are two linearly independent unit vectors of the hexagonal lattice and *n* and *m* are integers. The unique electronic properties of SWCNTs depend on the chiral vector and are divided into metallic ($n-m=3k$) and semiconducting ($n-m\ne 3k$), where *k* is an integer. Semiconducting SWCNTs are mainly used in optical applications and are selected according to the applied wavelength band. We purchased super-purified SWCNTs produced in the HiPco$^{TM}$ (high-pressure carbon monoxide) process from NanoIntegris. The diameter of individual SWCNTs was between 0.8 and 1.2 nm (mean diameter: 1.0 nm), which is suitable for mode locking of EDFLs operating in the 1550 nm wavelength band.

Polyvinyl alcohol (PVA), poly methyl methacrylate (PMMA) and sodium carboxymethylcellulose (NaCMC) are commonly used for the host polymer of SA films but have several limitations. The low glass transition temperatures of PVA (85 $^{\circ }$C) and PMMA (105 $^{\circ }$C) limit the thermal endurance, and NaCMC is vulnerable to moisture. Polydimethylsiloxane (PDMS) was selected because of its high thermal stability below 350 $^{\circ }$C and moisture resistace. PDMS was purchased from Dow Corning, and chloroform (solvent of PDMS) was purchased from Sigma Aldrich.

## Method

### CNT-Polymer SA Preparation

Preparation of a uniform and stable dispersion of CNTs is the first step in the fabrication of any CNT-based composite material due to their strongly aggregated state. Direct manual mixing of CNTs and a polymer matrix mostly results in poor distribution of CNTs. Agglomeration of CNTs generally causes severe problems in composite performance because it limits mechanical, thermal and optical properties by hindering the flow of energy through the interconnected network of the polymer. We selected the common solvent chloroform (${\text {CHCl}_{3}}$) to facilitate the integration of CNTs and PDMS. Even though several solvents are available for PDMS, chloroform was chosen because of the relative ease of evaporation at a later stage of the experiment. The limited chemical reactivity of functionalized CNTs (fCNTs) enables them to unbundle themselves during reaction and establish bonding interactions with the active chemical groups in the polymer matrix, thereby ensuring a proper and uniform dispersion of the nanofillers.

**Fig. 1:**
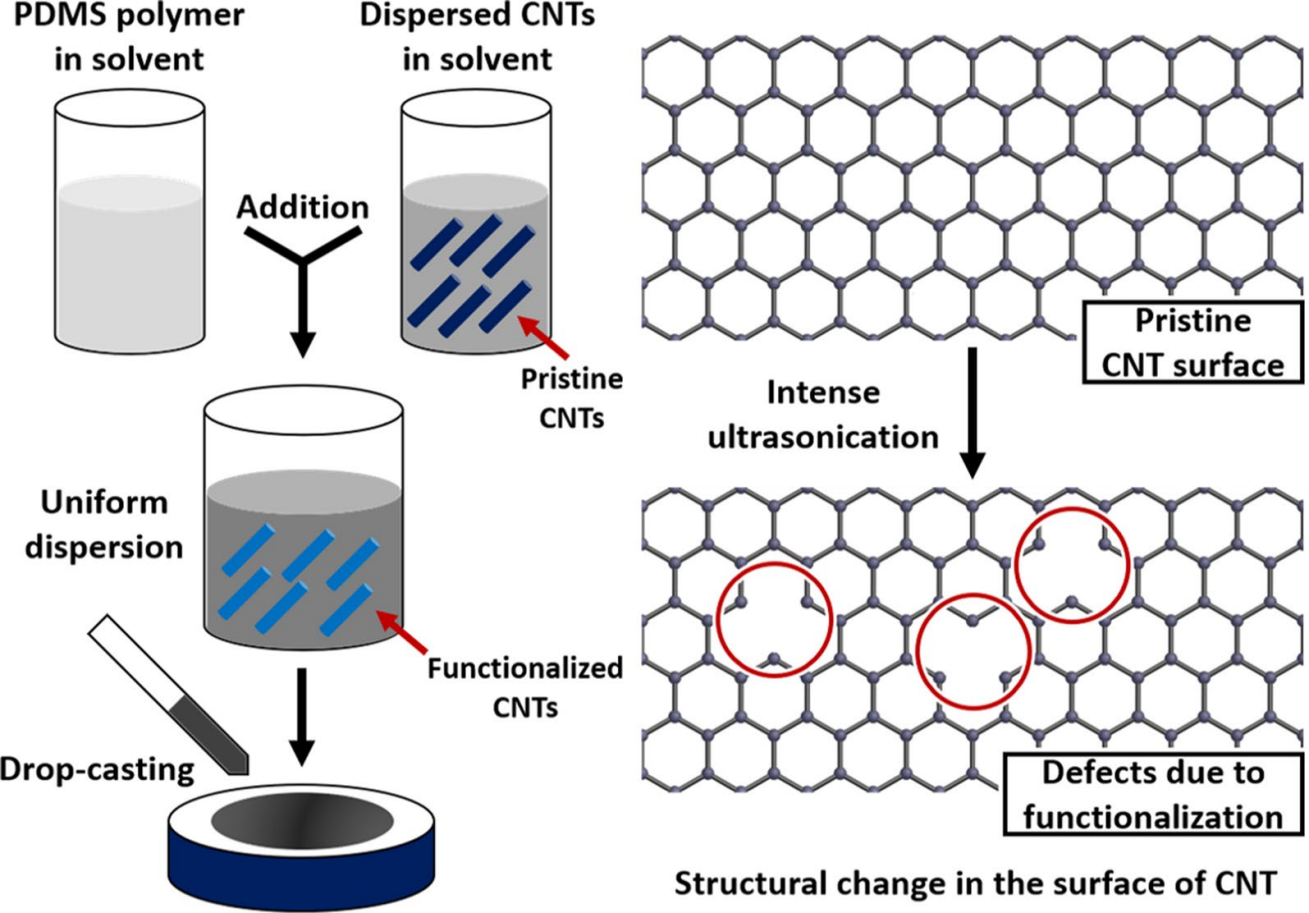
Schematic representation of partial functionalization of SWCNTs and overall film-type SA preparation process

A carefully calibrated ultrasonication-assisted procedure induces partial surface modification (or functionalization) of the CNTs by transforming the sp$^{2}$ hybridized atoms in the network into the sp$^{3}$ configuration. The electron-rich sites of the sp$^{3}$ network enable efficient unbundling, disruption of the $\pi -\pi$ attachment and, finally, uniform attachment to the polymer matrix. This phenomenon eliminates the possibility of agglomeration by nanofillers, which is the most common problem observed in composite materials [22]. Figure 1 shows a schematic diagram of the preparation procedure. We ultrasonicated 5 mg of CNTs in chloroform to prepare a uniform and stable dispersion. We used 350 W probe ultrasonication (ULSSO HITECH, ULH700S) for approximately 3 h to introduce surface defects and minimize the degree of agglomeration of the CNTs. After the preparation of the CNT dispersion, we added it to 1 g of PDMS with constant stirring for another 60 min and then ultrasonicated the mixture for 30 min. We added a hardener to the CNT-PDMS mixture at an amout equaling 1/5th of that of the polymer and stirred again for 10 min. We dropped the mixture into a glass petri dish in a controlled manner and left it to settle for 30 min at 90 °C. Then, another layer of polymer mixture was added using a similar procedure, and the double-layered film was left to cure for 90 min at 90 °C.

### Fiber Laser Setup

We set up a femtosecond fiber laser to test the mode locking capability of the fSWCNT-PDMS SAs. In soliton mode locking, when the soliton pulse goes through the fiber cavity, no temporal or spectral changes in the pulses occur as a result of the combined effects of chromatic dispersion and nonlinearity (self-phase modulation). Even though soliton lasers have some limitations in practical applications due to spectral sideband generation, among others issues, they are relatively stable and easy to control.

**Fig. 2:**
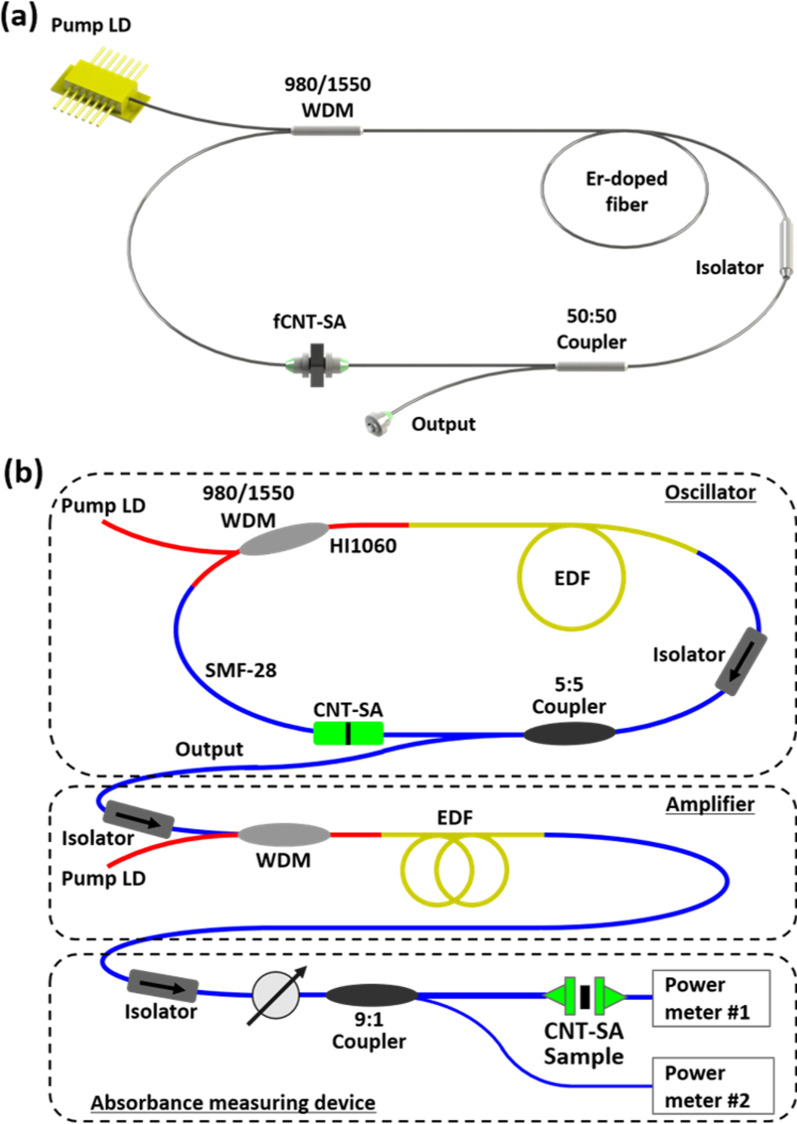
Schematics of the **a** passively mode-locked EDFL setup and **b** nonlinear absorbance measuring system

The laser setup is shown in Fig. 2a. The optical fiber cavity is composed of a 980/1550 wavelength-division multiplexer (WDM), an isolator, a 50:50 coupler and an erbium-doped fiber (EDF). It was designed in a ring configuration, and all single-mode (SM) fibers were fusion spliced. To operate the fiber laser in the soliton mode-locked regime, negative net dispersion is required. Therefore, the lengths of the positively dispersive EDF and negatively dispersive SMF-28 and HI1060 were adjusted. The length of the EDF (0.033 ps$^{2}$/m at 1550 nm) was 93 cm, SMF-28 ($-0.023$ ps$^{2}$/m at 1550 nm) was 159 cm and HI1060 ($-0.007$ ps$^{2}$/m at 1550 nm) was 16 cm. The total intracavity group velocity dispersion (GVD) was $-0.007$ ps$^{2}$/m, and the total length of the cavity was 268 cm.

Erbium (Er) belongs to the group of rare earth materials and was used in the form of the trivalent ion Er$^{3+}$ as the laser-active dopant of the gain medium. The EDF was used for the lasing medium, and it was laser pumped by an external energy source. The WDM connects the pump laser diode (LD) to the laser cavity with the HI1060, which delivers both a 980 nm pump source and a 1550 nm pulsed laser. The SA film was inserted between the FC/APC connectors located between the WDM and coupler.

To evaluate the performance of SAs, two kinds of representative indicators, the modulation depth and nonsaturable loss, should be determined. The modulation depth is the maximum change in absorption (or reflection) of SAs that emerges due to incident light. This is the most important parameter: the laser cannot be mode locked in the case of a modulation depth lower than a sufficient value. In the soliton regime, a moderate modulation depth can be used, but in the case of other regimes (stretched pulse, etc.), a very high value is required for mode locking because of the high instability. The nonsaturable loss is typically an unwanted factor of an SA, and its value is the absorption rate when the SA is saturated at high intensity. In the case of a thin film SA, an excessively high concentration of nanomaterial and a high thickness of the film lead to a high value of the nonsaturable loss.

In general, SAs tend to exhibit increasd transmittance under high-intensity light. Therefore, to determine the saturation absorption characteristics, the power incident on the SA must be changed and how the absorption rate changes accordingly measured. A schematic diagram of the nonlinear absorbance measuring system is presented in Fig. 2b. This system was designed to measure optical properties by the power-scan (p-scan) method, which measures the transmittance of the SA according to the incident optical intensity. There are three different parts in the system: an oscillator, an amplifier, and an absorbance measuring device in the system. All the three parts are connected by SMF-28 and isolator is located between each to maintaining forward direction delivery. The entire system was developed in the form of an all-fiber system. The configuration of the fiber oscillator is the same as in Fig. 2a except for the length of each optical fiber. It generates femtosecond pulses in the wavelength band in which the SA will be operated. In the fiber amplifier, the EDF is forward pumped by an additional laser diode. The erbium ions are excited into metastable states and amplify the seed laser via stimulated emission. The low-power seed laser emitted from the oscillator passes thorough the fiber amplifier, and very high-intensity pulses enter the absorbance measuring device. The intensity of light incident on the SA is controlled through a variable attenuator (Thorlabs, VOA50), and light is divided in a ratio of 9:1 by an optical fiber coupler. Ninety percent of the light passes through the SA, and the power ofthe transmitted light is measured. The power of the remaining 10% of the light is measured directly by another power meter. Since the optical power emitted from the 90% terminal of the coupler can be inferred through the optical power measured at the 10% terminal, the light incident on the SA can be identified. The transmittance of the SA can be calculated using this value and the optical power of light transmitted through the SA. The two power meters are connected to a computer that enables real-time monitoring.

## Results and Discussion

### Structural Analysis of the CNTs and fCNTs

Structural analysis of the CNT and fCNT samples was carried out by applying XRD and Raman spectroscopy techniques. XRD was helpful in ascertaining the degree of the difference in the crystallinity of the samples. We analyzed the extent of damage or reorganization of structural bonding in the CNT walls as a result of intensive ultrasonication via Raman spectroscopy.

**Fig. 3:**
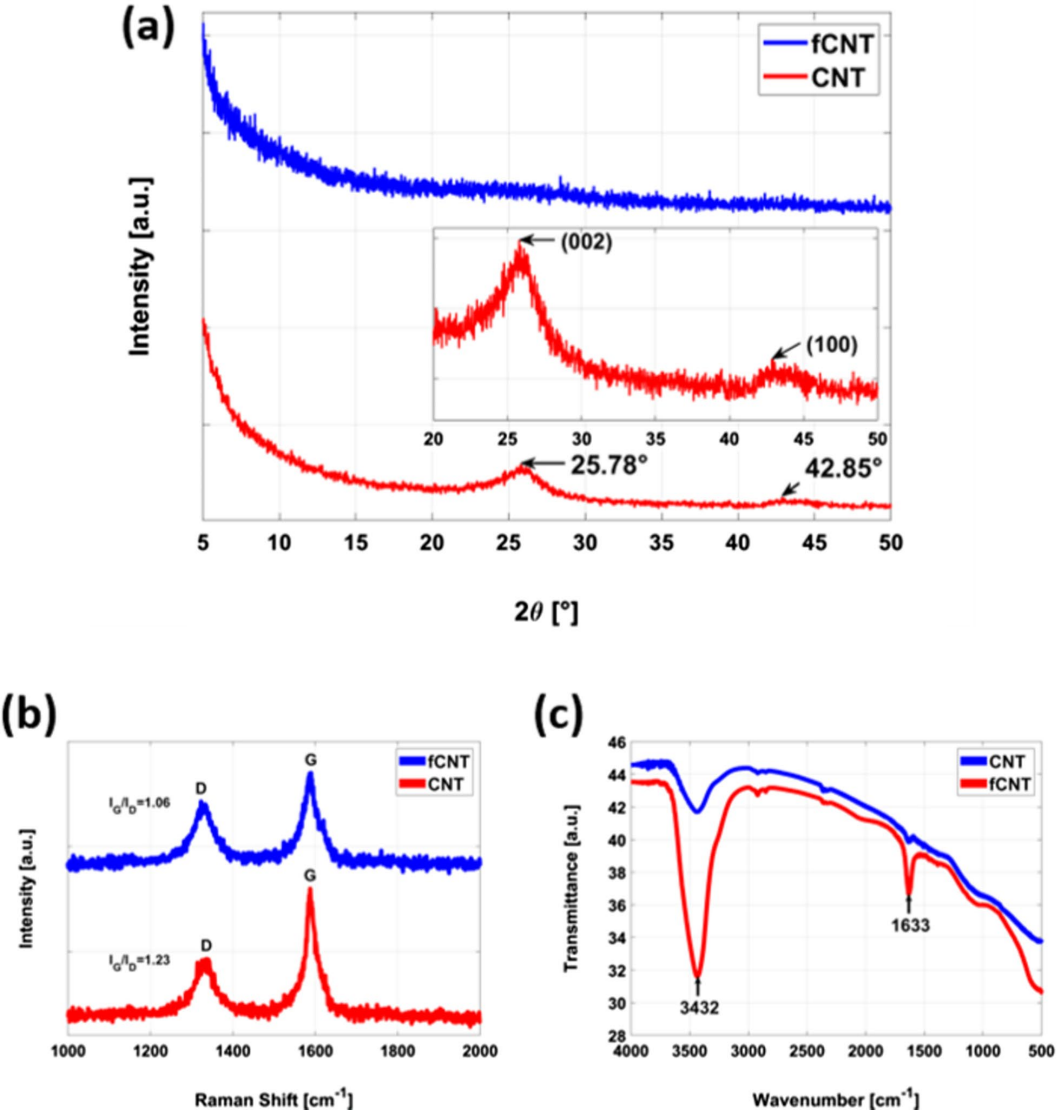
**a** Comparison of XRD results for SWCNT and fSWCNT powders (inset: enlarged graph of SWCNT data in the 20–50° range). Comparison of **b** Raman analysis and **c** FTIR results of SWCNT and fSWCNT powders

The typical crystalline nature of the CNT structure results in a strong peak at $\sim$26° in the XRD spectrum corresponding to the (002) plane, which generally indicates interlayer distance of approximately 0.34 nm (by applying Bragg’s law) [23]. The (100) peak for CNTs reflect the hexagonal graphite structure [24]. The XRD result of CNTs is shown at the bottom of Fig. 3a. In the case of SWCNTs, the (002) peak is slightly shifted toward the larger interlayer distance than in the case of MWCNTs, and is broad and asymmetric. These results demonstrate that the (002) peak reflecting the intertube distance (outer-wall contacts) and the broadening is caused by curvature effects due to the cylindrical shape [25]. The (100) peak is shown in the inset to appear at $\sim 43^{\circ }$, which corresponds to a spacing of $\sim$0.2 nm [26]. When the crystallinity is disturbed in graphitic materials, this results in the collapse of the C=C bonding integral to $\text {sp}^{2}$ hybridization and creates $\text {sp}^{3}$ bonding [27]. These structural disturbances can be initiated either by disturbing the bonds with outside passive forces or by inserting foreign functional groups [28]. In this case strong ultrasonication in the solvent resulted of structural modification in the CNT walls, which caused disappearance of the (002) peak [29]. The change of (002) peak according to the time of ultrasonication can be confirmed in Additional file 1. In the XRD pattern of 1-hour sonicated sample, (002) peak becomes broad and moves toward the direction of low angle. And in the XRD pattern of 2-hour sonicated sample, (002) peak becomes even more broader and moves further toward the direction of increasing intertube distance. Lastly, we confirmed that (002) peak eventually disappeared in the XRD pattern of 3-hour sonicated sample (Additional file 1: Figure S1).

We obtained further confirmation of this structural modification in the CNT samples by the Raman spectroscopic results in Fig. 3b. The Raman spectra of CNT and fCNT samples consist of two main peaks called the defect peak (D peak) and graphitic peak (G peak). While the G peak represents sp$^{2}$ hybridized C=C bonding formation, the D peak typically denotes disordered graphitic structures or sp$^{3}$ hybridized carbon atoms [30]. The CNTs showed D and G peaks at 1336 cm$^{-1}$ and 1588 cm$^{-1}$, respectively, while the peaks were at 1323 cm$^{-1}$ and 1590 cm$^{-1}$ for fCNTs in the same order. The ratio of the peak intensites (I$_{G}$/I$_{D}$) reveals the extent of the structural integrity of the samples [31]. Applying passive external forces such as rigorous ultrasonication causes stress on the C=C bonds that induces them to open up, creating electron-rich carbon centers and bringing considerable damage to the CNT wall [32]. We calculated the relative $I_{G}/I_{D}$ ratios of the CNT and fCNT samples after ultrasonication, which showed increased relative D peak intensity in the latter sample. The overall $I_{G}/I_{D}$ ratios were 1.23 and 1.06 for the CNTs and fCNTs, respectively, indicating structural modification because of mechanically induced functionalization. Since we wanted to largely preserve the innate characteristics of the CNT structure, we call this process a partial functionalization in which structural damage was initiated with the sole purpose of including a uniform bonding interaction between the nano-filler and the polymer matrix.

FTIR spectrosocpy was carried out to confirm that functional groups were attached to SWCNTs during ultrasonication in chloroform. The results measured through the KBr sample pellet are shown in Fig. 3c. Chloroform is mainly physiabsorbed into SWCNTs, but there is also the possibility of partially forming the SWCNT-$\text {CCl}_{2}$ complex [33]. In previous research, SWCNT-$\text {CCl}_\text {2}$ showed a peak around 2800 cm$^{-1}$, but in this result, it can be confirmed that there is no related functional group because the corresponding peak is absent. The characteristic absorption band for the C=C bond stretching vibrations appearing at about 1600 cm$^{-1}$. In general, SWCNTs show a weak IR spectrum because the difference in charge state between carbon atoms is small. In functionalized SWCNTs, generation of induced electric dipoles is enhanced and the size of the absorption peak increases as the symmetry of the nanotube is broken. It can be seen in Fig. 3c that the surface modification caused by a partial defect increased the peak located at 1633 cm$^{-1}$. The broad absorption band around 3400 cm$^{-1}$ can be caused by oxidation in the process of purification by the manufacturer, and is also greatly affected by moisture. The reason that fSWNCT shows increased absortpion at 3432 cm$^{-1}$ is considered to be because the defect site absorbed moisture during drying for FTIR measurement. It is considered that the increase in the absorption band centered at 3432 cm is due to the absorption of moisture by the defective site during the drying process.

### Analysis of SA Thin Films

#### Structural and Chemical Analysis

**Fig. 4:**
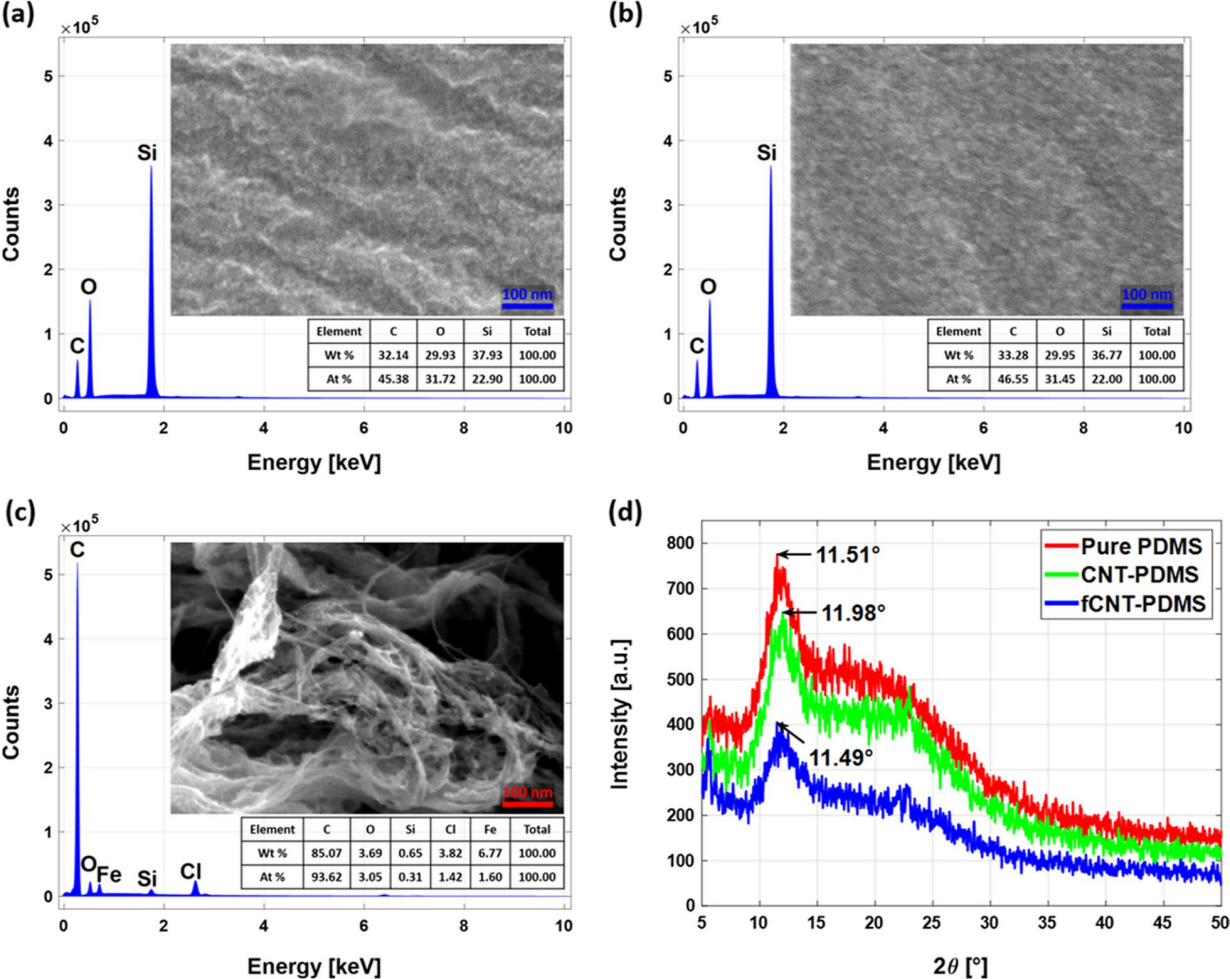
SEM images and EDS results for **a** SWCNT-PDMS, **b** fSWCNT-PDMS composite films and **c** SWCNT powder. **d** Comparison of XRD results for pure PDMS and SA thin films

Generally, CNTs are supplied in powder form and most of them are bundled as shown in Fig. 4c. Without functionalization, CNTs remains aggregated, and the degree of dispersion is inferior, as shown in Fig. 4a. Figure 4b shows uniformly distributed CNTs without severe aggregation in the fSWCNT-PDMS composite film. EDS (energy dispersive spectroscopy) analysis shows 6.8 wt% of residual iron (Fe) catalyst in powdered CNTs. Peaks for the elements of PDMS, i.e., oxygen (O), silicon (Si) and carbon (c), are shown in the data for SWCNT-PDMS composites which is in good agreement with previously reported PDMS matrix data [34].

We performed XRD studies to analyze the structural features of the polymeric SA films, and Fig. 4d shows the results. Pure PDMS shows a characteristic halo at 11.51°. The SWCNT-PDMS and fSWCNT-PDMS samples show sharp peaks at 11.98° and 11.49°. When mixed with fSWCNTs, the intensity of the halo peak from PDMS and SWCNT-PDMS decreases, but the peak remains at a similar position, possibly due to uniform mixing and superimposition with the fSWCNT peak [35].

**Fig. 5:**
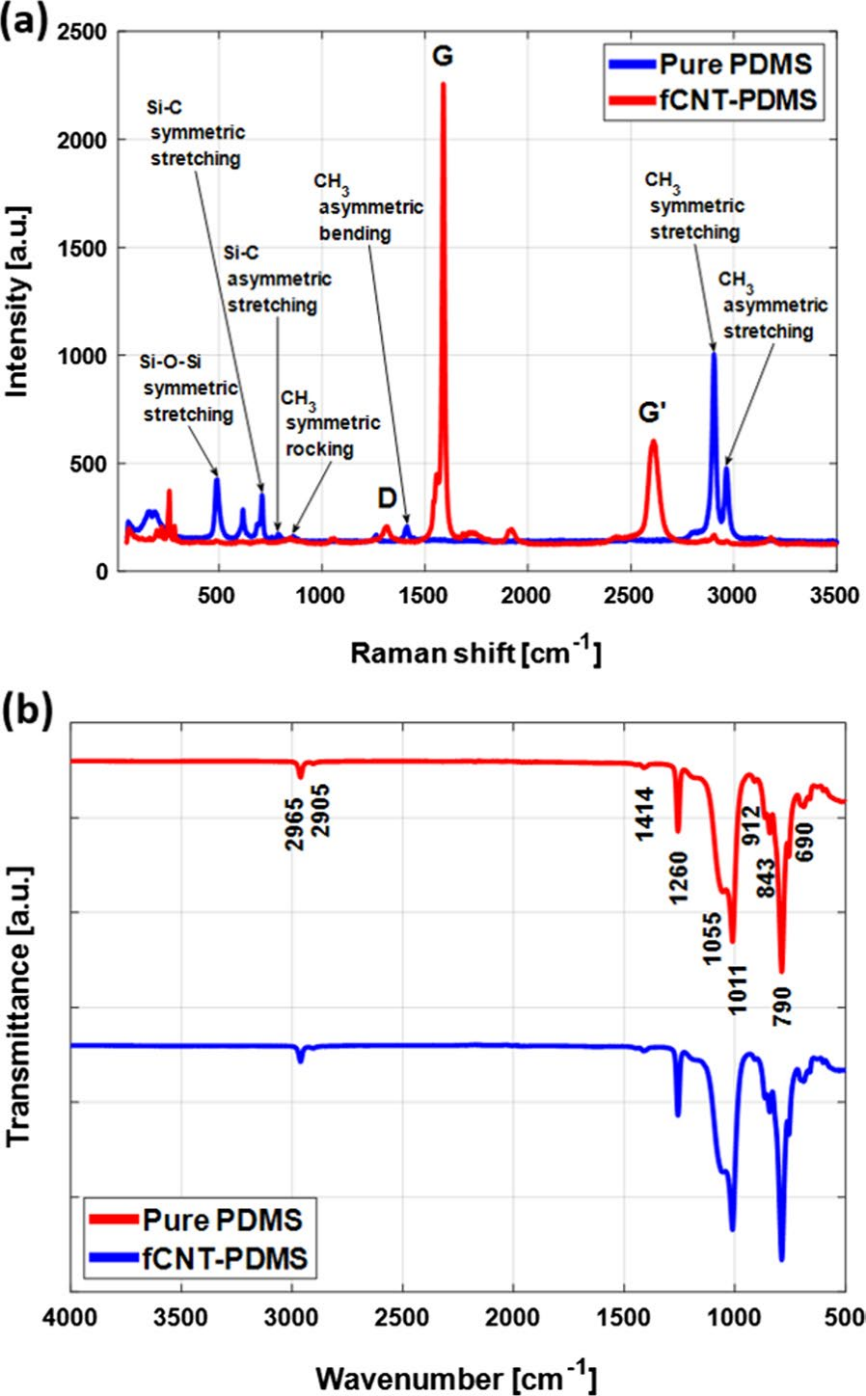
Comparison of **a** Raman analysis and **b** FTIR results of pure PDMS and fSWCNT SA thin films

We performed Raman spectroscopy on the samples consisting of pure PDMS SA films and composite SAs to ascertain the structural changes, if any, as a result of nanofiller addition. In Fig. 5a, the SA films show peaks typical of PDMS, but in the composite SA films, there are extra peaks due to the fSWCNT addition. The separate and distinct peaks at 491 cm$^{-1}$, 708 cm$^{-1}$, and 785 cm$^{-1}$ indicate the presence of Si–O–Si symmetric stretching, Si–C symmetric stretching and Si–C asymmetric stretching, respectively. We identified $\text {CH}_{3}$ symmetric rocking and asymmetric bending vibrations with clear peaks at 860 cm$^{-1}$ and 1413 cm$^{-1}$, respectively. The peaks at 2904 cm$^{-1}$ and 2963 $\text {cm}^{-1}$ represents $\text {CH}_{3}$ symmetric stretching and $\text {CH}_{3}$ asymmetric stretching vibrations, respectively [36]. The changes in the intensities and the broadening of some characteristic peaks along with the emergence of prominent D, G and G$^\prime$ peaks at approximately 1317 $\text {cm}^{-1}$, 1589 $\text {cm}^{-1}$ and 2610 $\text {cm}^{-1}$ confirm the presence of and attachment between the polymer and nanofiller.

FTIR spectroscopy was carried out to analyze the presence of different functional groups in the pure PDMS and fSWCNT-PDMS SA films, and the results are shown in Fig. 5b. The different functional groups present in the PDMS and fSWCNTs are mainly instrumental for the bonding interactions between the nano-filler and the polymer matrix, thereby affecting the overall properties of the final SA films. The absence of any major or minor peaks at 3300 $\text {cm}^{-1}$ and 1700 $\text {cm}^{-1}$, which typically indicate the presence of hydroxyl and carboxylic groups, confirms the total evaporation of acetic acid residues from the composite films due to the curing process. The peaks at 2905 $\text {cm}^{-1}$ and 2965 $\text {cm}^{-1}$ are indicative of symmetric and asymmetric $-\text {CH}_{3}$ stretching in $\equiv \text {Si}-\text {CH}_{3}$ from PDMS. The major peak at 843 $\text {cm}^{-1}$ denotes the rocking peak of stretching bands originating from Si–C bonds. The sharp peak at approximately 790 $\text {cm}^{-1}$ corresponds to the rocking vibration of the Si–$(\text {CH}_{3})_{2}$ group, and the peak at 690 $\text {cm}^{-1}$ is related to Si–C stretching. The peaks at approximatley 1414 $\text {cm}^{-1}$ and 1260 $\text {cm}^{-1}$ are related to asymmetric and symmetric stretching of C–H bond from Si–$(\text {CH}_{3})_{2}$. Multiple peaks at approximately 1011 $\text {cm}^{-1}$ and 1055 $\text {cm}^{-1}$ identify the stretching of Si–O–Si from the long chain structure of PDMS [37]. The peak intensity of the films containing fSWCNTs appears to slightly decrease with better dispersion of the SWCNTs in the films, possibly due to the formation of Si-C bonds between the PDMS structure and fSWCNTs. We did not find any significant difference between the pure PDMS film and the fSWCNT-PDMS film apart from some difference in intensity at approximately 912 $\text {cm}^{-1}$, which is related to Si–H bending [38].

#### Thermal Stability

**Fig. 6:**
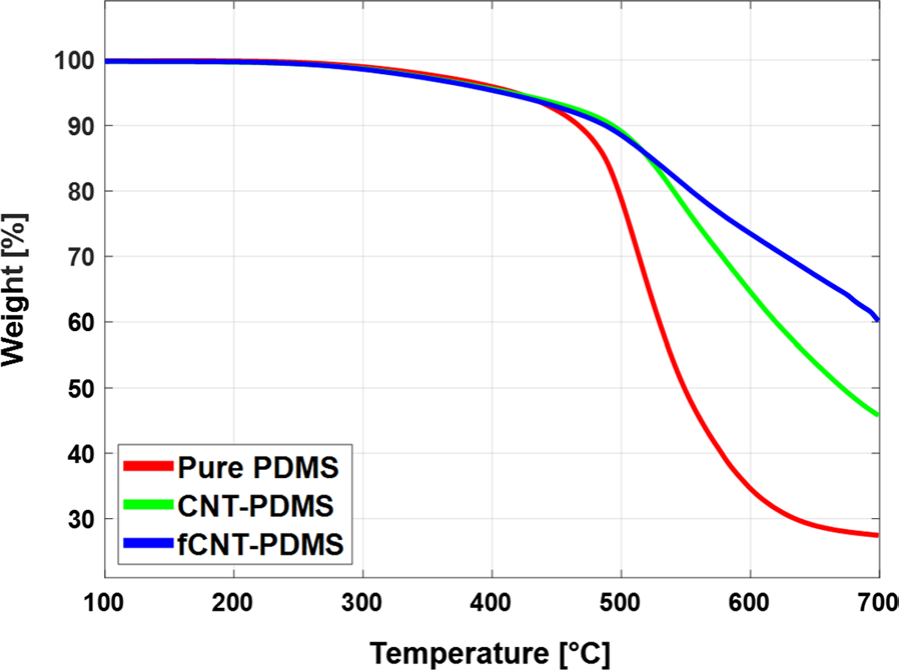
Comparison of TGA results of pure PDMS and film-type SAs

Figure 6 shows the TGA results of the SA samples. Since ultrashort pulse laser operation generates considerable electric power in the laser cavity, the SAs inevitably suffer thermal damage over time. Before placing the SAs inside the laser cavity, we tried to determine their thermal threshold by TGA. In this experiment, small samples of SA thin films were placed in nitrogen $(\text {N}_{2})$ environment and heated. The rate of temperature increase was fixed at 10 °C/min, and the temperature was recorded from 100 to 700 $^{\circ }$C. This experiment helped identify the thermal degradation threshold by measuring the total mass of the samples with respect to the increasing temperature as well as their end mass after the experiment was completed. The thermal stability is directly proportional to the extent of uniform attachment between the polymer and the nanofiller. PDMS starts to decompose into its organic and inorganic parts at approximately its boiling temperature of 420 °C. During this pyrolytic process, it gives SiOx ($x = 1, 2$) compounds and carbon ash while losing mass weight at a significant rate [39]. In this case, the CNT-PDMS and fCNT-PDMS thin films exhibit similar onset temperatures for the initiation of structural damage. While the fCNT-PDMS SA films retained almost 60% of the initial mass, the PDMS SA and CNT-PDMS films retained only 27% and 45% of the starting mass, indicating significant structural cohesion [40]. The covalent bonds between the fCNTs and PDMS matrix due to both the unique nano filler properties and the double layer fabrication process prevented total dissolution of the composite structure [41].

### Damage Test

We put the composite SA samples through rigorous damage testing experiments. Since a continuous wave laser is more likely to cause severe thermal damage in thin film SAs, we employed it for this purpose. The damage testing conditions featured high-powered fiber laser operation at a significantly elevated capacity compared to that required in ordinary conditions for an extended amount of time. The rationale behind running the fiber laser under these stringent conditions was to ensure significant damage to the SA film samples. The experiment lasted continuously for 12 h. The samples showed sustained damage resistivity over a wide range from 4 to 13 mW before the degradation accelerated.

**Fig. 7:**
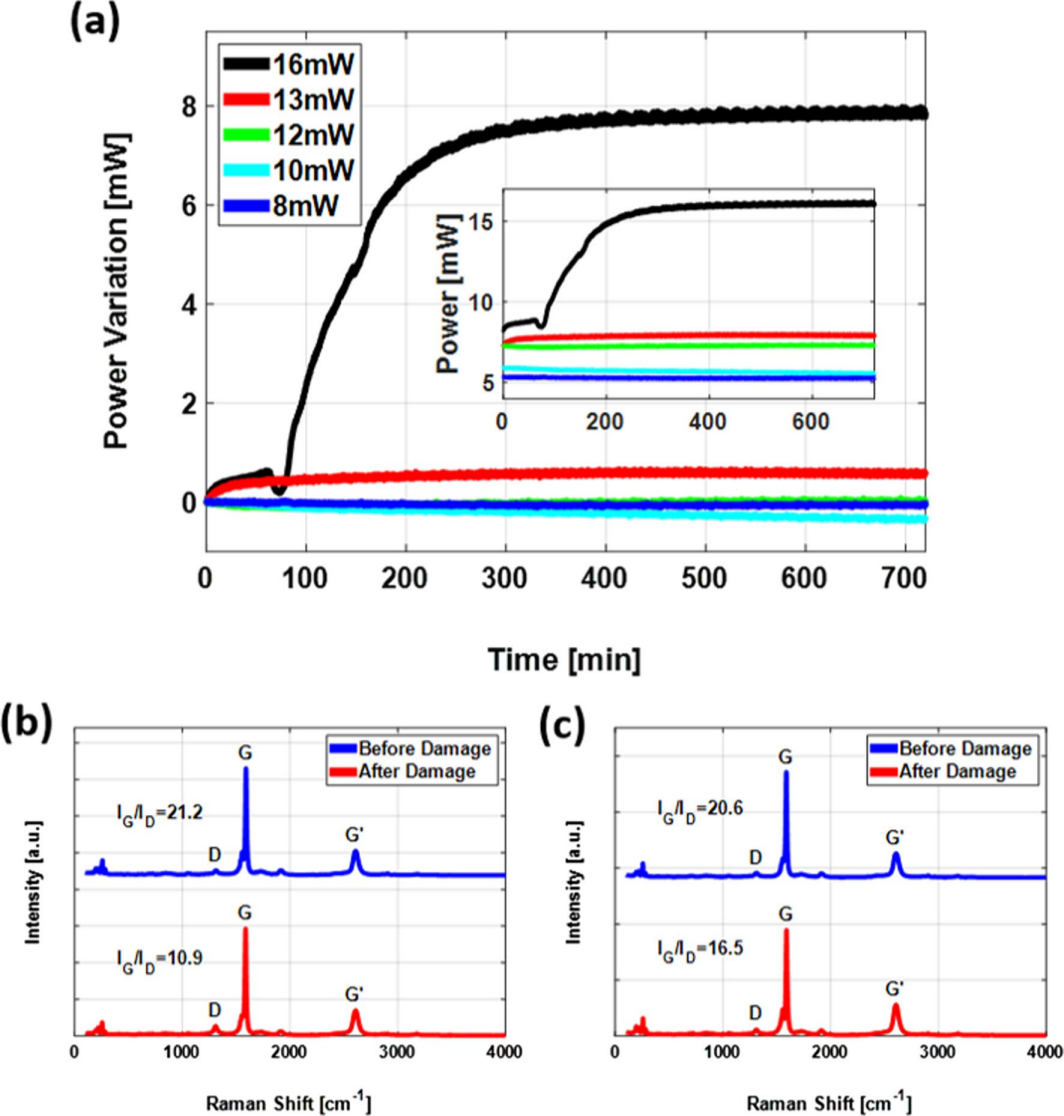
**a** Variation in the output power during the damage test of a film-type SA (inset: output power of light after transmission through the SA). Comparison of damage test results of **b** SWCNT-PDMS and **c** fSWCNT-PDMS thin films

Figure 7a shows the variation in the optical power of the light transmitted through the SA samples for 12 h. There was almost no change in the absorption of the fSWCNT-PDMS SA for incident light power of 8 mW or less. When the SA is exposed to a power of 10 mW, the absorption is very slightly increased. This occurs due to graphitization of SWCNTs and increases the temperature of the SA. As a result, SWCNTs are burned out, and the absorption of the SA is decreased due to the lowered SWCNT concentration. At 12 mW incident power, graphitization and burning out occur simultaneously, and the change in absorbance is balanced. The amount of change is very small and stability is maintained under 12 mW power. The absorbance of the SA decreases noticeably at 13 mW incident power. This is caused by burning out of SWNTs in limited areas where dispersion is not perfectly achieved. The fCNT-PDMS SA shows significantly improved stability compared to previous research [42], in which the burning out stages started at 5 mW and the damage threshold was 8 mW.

We analyzed the damage-tested samples by Raman spectroscopy and compared their extent of structural integrity with the Raman result. The results are presented in Fig. 7b, c. We identified 13 mW as the threshold for the samples and measured the ratio of the intensities of the graphitic peak and the defect peak in the Raman spectrum at this point to establish the overall structural integrity of the sample. We tested a different sample prepared following exactly the same process consisting of SWCNTs without functionalization at 13 mW compared its results with those of our original test samples. After analysis, the extent of structural damage in the fSWCNT-based SA sample became clear by comparing the Raman spectra [43]. As shown in Fig. 7b, the $I_{G}/I_{D}$ ratio of the fSWCNT-PDMS SA film was 20.6 before commencing the damage test and remained at 16.5 after the damage test, retaining over 80% of the initial structural composition. In contrast, without functionalization, the SWCNT-based PDMS SA retained only approximately 51% of the initial structural composition after the 12 h damage test, as shown in Fig. 7c.

### Laser Performance

A performance test of fSWCNT-PDMS SA films was conducted on the sample that had been subjected to the 12 h damage test at 12 mW incident power. An SA is an optical component whose absorption rate decreases as the intensity of light passing through it increases. The saturation of the absorption rate of the SA can be phenomenologically explained based on a two-level electronic model. The absorption rate is commonly modeled as

2 $$\begin{aligned} \alpha (I)=\alpha _0\left( 1+\frac{I}{I_{s}}\right) ^{-1} \end{aligned}$$

where $\alpha (I)$ is the intensity-dependent absorption coefficient of the SA, $\alpha _0$ is the linear absorption coefficient at low intensity, *I* is the light intensity and $I_s$ is the saturation intensity of the SA [44]. The saturation absorption model in Eq. (2) is a model that calculates the absorption rate for an ideal SA, and when the incident light intensity is infinite, the absorption rate converges to zero. However, in reality, an intrinsic loss inevitably occurs due to elastic scattering, etc., which is related to the nonsaturable absorption coefficient ($\alpha _{ns}$). Therefore, the absorption model of an actual SA can be expressed as

3 $$\begin{aligned} \alpha (I)=\alpha _0\left( 1+\frac{I}{I_{s}}\right) ^{-1}+\alpha _{ns} \end{aligned}$$

**Fig. 8:**
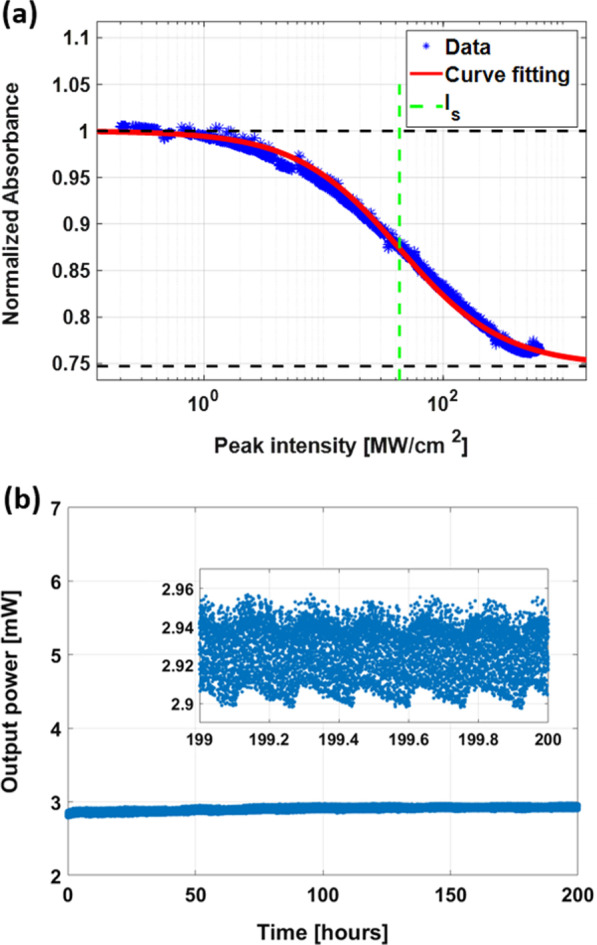
**a** Normalized saturable absorption of the fSWCNT-PDMS SA. **b** Stability test of the passively mode-locked EDFL for 200 h (inset: enlarged graph for 1 h)

The saturation absorption characteristics of the SA samples were measured with a nonlinear absorbance measurement system that delivered $\sim$300 fs pulses at a central wavelength of 1550 nm. The nonlinear transmittance increases from 11.6% to 22.0%. By fitting the measured data to Eq. (3), the saturation intensity ($I_{s}$) is 43 MW/$\text {cm}^{2}$ (0.37 mW in average power) and the modulation depth ($\Delta \alpha$) is 25.3%, as shown in Fig. 8a. In the measured data, some spikes occurred when the measuring range of the optical power meter was changed.

Figure 8b shows the long-term stability of the fSWCNT-PDMS SA, and it ensures durable operation of mode-locked fiber lasers. The inset shows the enlarged figure for 1 h. The small perturbations and periodic changes are thought to be caused by other factors, such as the temperature control of the laser diode.

**Fig. 9:**
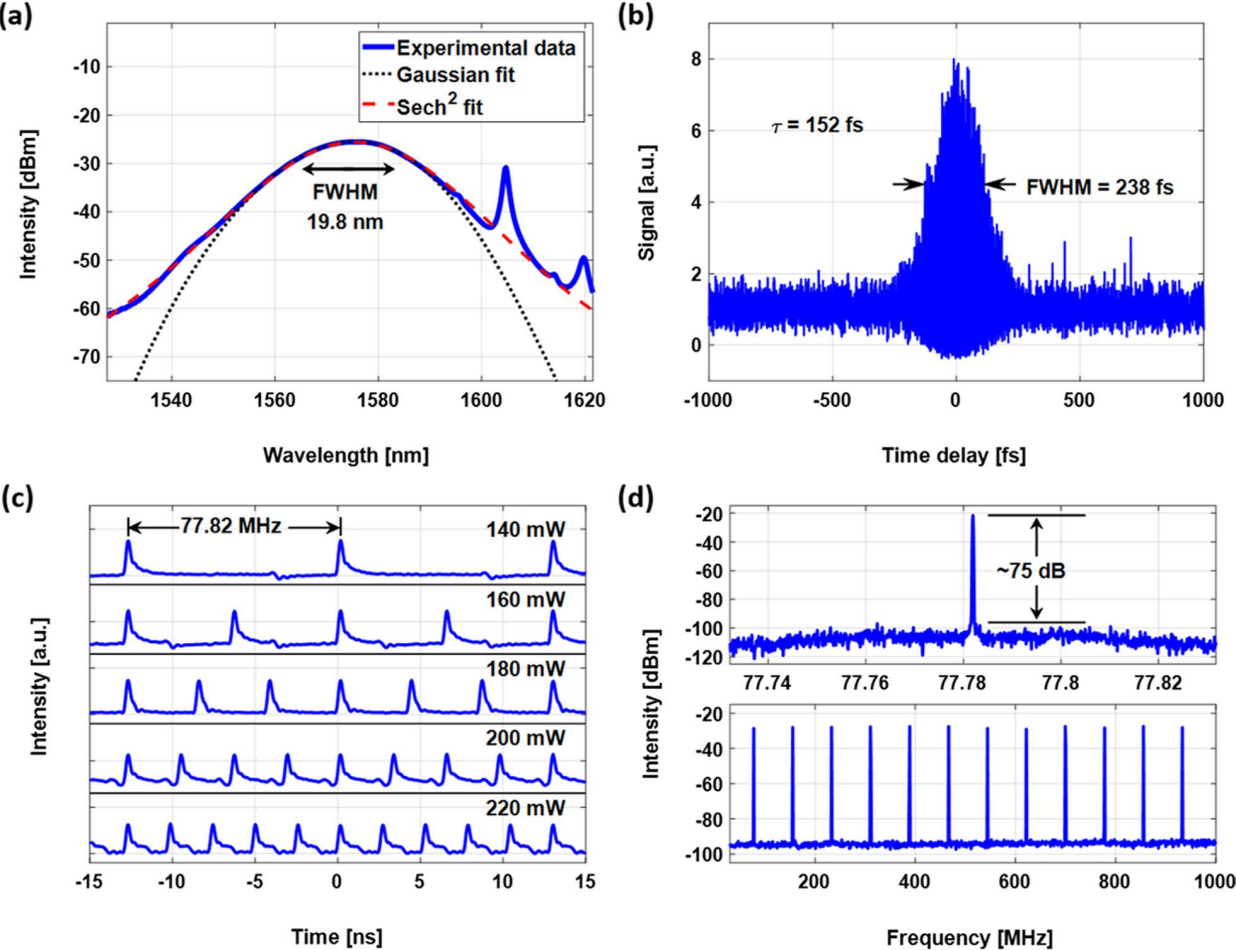
Pulse output properties of the laser: **a** optical spectrum, **b** autocorrelation trace, **c** pulse trains under different pump powers, **d** RF spectrum at the fundamental repetition rate with a 100 Hz resolution bandwidth (top), and RF spectrum with 1 GHz with 30 kHz resolution bandwidth

The soliton fiber laser demonstrated self-starting single-pulse mode locking between 90 and 140 mW pump power. Figure 9 shows the optical spectrum, autocorrelation trace, pulse train and radio frequency (RF) signal spectrum from the EDFL setup. The average output power is 2.9 mW when it operates at a pump power of 140 mW. The existence of Kelly sidebands, a characteristic of soliton pulses, is shown in Fig. 9a. The optical spectrum exhibits a typical soliton spectrum shape (sech^2^) with a central wavelength of 1576 nm. The full-width at half-maximum (FWHM) bandwidth is 19.8 nm. Figure 9b shows the autocorrleation trace. The FWHM pulse duration is 238 fs, and assuming a sech^2^ pulse shape, the inferred pulse duration is 152 fs. The time-bandwidth product is 0.373 which means that pulses are close to transform-limited. The pulse train is shown in Fig. 9c, and the repetition rate is 77.82 MHz. Multiple pulses are obtained with equidistant spacing at high pump powers and quintuple pulsing is maintained up to 340 mw. The RF signal spectrum around the fundamental repetition rate is shown in Fig. 9d. From the signal-to-noise ratio (SNR) of $\sim$75 dB, we can verify the stability of pulsed laser operation with low amplitude fluctuation and timing jitter. Compared with the mode-locked fiber laser using the CNT-PDMS composite of the previous research, it can be confirmed that the spectral bandwidth, pulse duration, and SNR are improved based on the superior SA performance [45].

## Conclusion

Partially functionalized SWCNTs were uniformly incorporated within a PDMS polymer matrix, and fSWCNT SAs showed high damage resistance with enhanced durability. The mechanical functionalization process solved the problem of CNT aggregation without using any surfactant or effecting chemical functional groups, both of which compromise the inherent nonlinear characteristics in conventional methods. The SAs demonstrate superior thermal stability and damage resistance during TGA and rigorous damage tests, so they ensure high-power ultrashort laser operation by virtue of their superior material characteristics and preparation procedure. Stable mode locking was acheived by placing the fSWCNTs into an EDFL. The ultrashort pulse trains exhibited a 77.82 MHz repetition rate, a 152 fs pulse duration and an SNR of 75 dB in single pulse generation. Stable femtosecond laser operation was also demonstrated for over 200 h, so the proposed fSWCNT SA provides a promising solution for laser applications that require ultrashort pulsed lasers with long-term durability. We demonstrated new approaches for developing damage-resistant polymer SA films and comprehensive analyses for self-standing SA films. Various related studies on SAs that use other low dimensional materials and photonic devices for pulsed fiber lasers with high output energy are expected based on this work.

## Experimental Section

### Fiber Components

A fiber Bragg grating-stabilized 980 nm pump laser diode (JDSU, S30) connected to a tabletop laser and a Peltier controller (OsTecch, ds11-t85) was used as the laser source. Three kinds of SM optical fibers, SMF-28, HI1060 (high-index) and ER80-4/125 (erbium-doped fiber for 980 nm pumping with emission at 1550 nm), were purchased from Thorlabs. All SMF-28 (transmission from 1260 to 1650 nm), HI-1060 (transmission from 980 to 1650 nm) and ER80-4/125 (80±8 dB/m core absorption peak at 1530 nm) fibers had a diameter of 125 µm. The WDM combines two wavelengths (980 and 1550 nm) into a single fiber output, the isolator (1550 nm center wavelength) prevents reverse transmission of light, and the $1 \times 2$ coupler splits the light at a 50:50 ratio. Three fiber optic components, the WDM (WD9850BA), isolator (IO-H-1550) and coupler (TW1550R5A1), were purchased from Thorlabs. We used a cleaver (NorthLab Photonics, ProCleave SD/SFC) and a fusion splicer (UCL SWIFT, S3) for fiber processing.

### Laser Characterization

The fiber laser characterization consisted of measuring the spectral bandwidth, pulse duration, repetition rate and SNR of the output pulses. An indium gallium arsenide (InGaAs) photodetector (EOT, ET-3010) was used for optical signal acquisition. The spectral bandwidth was measured with an optical spectrum analyzer (Anritsu, MS9710C), and the pulse duration was measured with an autocorrelator (APE, Mini TPA/PD). The repetition rate was measured through a pulse train from an oscilloscope (InfiniiVision, DS07104A), and the SNR was measured through an RF signal from a spectrum analyzer (Advantest, R3477). The optical power was measured with two kinds of power meters: an integrating sphere photodiode power sensor (PM100D) and a compact fiber photodiode power sensor (S155C), which were both manufactured by Thorlabs.

### Polymer Composite Characterization

We analyzed the structural aspects of the CNT, fCNT and SA samples using XRD. Both high-resolution powder XRD (RIGAKU, SmartLab) and high-resolution thin film XRD (PANalytical, X’Pert-PRO MRD) were operated between 5° and 50° (2$\theta$). High-resolution Raman spectroscopic analysis (HORIBA, LabRAM HR Evolution Visible-NIR) was carried out with a 633 nm laser source. Chemical characterization typically involved FTIR spectroscopy using a Nicolet iS50 from Thermo Fisher Scientific Instrument. Thermal stability studies involved TGA experiments on a TG209 F1 Libra from Netzsch.

## Supplementary Information

**Additional file 1. Figure S1**. Change of (002) peak in XRD patterns according to ultrasonication time.** Figure S2**. Normalized saturable absorption of the SWCNT-PDMS SA.

## Data Availability

The data and materials in this article are fully available without restriction from the corresponding author on reasonable request.

## Abbreviations

SA: Saturable absorber
CNT: Carbon nanotube
SWCNT: Single-walled carbon nanotube
fSWCNT: Functionalized single-walled carbon nanotube
EDFL: Erbium-doped fiber laser
SESAM: Semiconductor saturable absorber mirror
SDS: sodium dodecyl sulfate
HiPco: High pressure carbon monoxide
PVA: Polyvinyl alcohol
PMMA: Poly methyl methacrylate
NaCMC: Sodium carboxymethylcellulose
PDMS: Polydimethylsiloxane
EDF: Erbium-doped fiber
SMF: Single mode fiber
GVD: Group velocity dispersion
Er: Erbium
LD: Laser diode
P-scan: Power-scan
G peak: Graphitic peak
D peak: Defect peak
FTIR: Fourier transform infrared
FWHM: Full-width half-maximum
SNR: Signal-to-noise ratio
SM: Single-mode
HI: High-index
WDM: Wavelength-division multiplexer
InGaAs: Indium gallium arsenide
RF: Radio frequency

## References

[1] Set SY, Yaguchi H, Tanaka Y, Jablonski M (2004). Ultrafast fiber pulsed lasers incorporating carbon nanotubes. IEEE J Sel Top Quantum Electron.

[2] Pegoraro AF, Ridsdale A, Moffatt DJ, Pezacki JP, Thomas BK, Fu L, Dong L, Fermann ME, Stolow A (2009). All-fiber cars microscopy of live cells. Opt Express.

[3] Jones DJ, Diddams SA, Ranka JK, Stentz A, Windeler RS, Hall JL, Cundiff ST (2000). Carrier-envelope phase control of femtosecond mode-locked lasers and direct optical frequency synthesis. Science.

[4] Debnath PC, Yeom D-I (2021). Ultrafast fiber lasers with low-dimensional saturable absorbers: status and prospects. Sensors.

[5] Chen Z, Wang Y, Lv R, Liu S, Wang J, Wang Y (2020). High-power erbium-doped fiber laser with a carbon nanotubes-doped sol-gel glass mode-locker. Opt Fiber Technol.

[6] Keller U, Weingarten KJ, Kartner FX, Kopf D, Braun B, Jung ID, Fluck R, Honninger C, Matuschek N, Der Au JA (1996). Semiconductor saturable absorber mirrors (SESAM’s) for femtosecond to nanosecond pulse generation in solid-state lasers. IEEE J Sel Top Quantum Electron.

[7] Okhotnikov O, Grudinin A, Pessa M (2004). Ultra-fast fibre laser systems based on SESAM technology: new horizons and applications. New J Phys.

[8] Mkrtchyan AA, Gladush YG, Galiakhmetova D, Yakovlev V, Ahtyamov VT, Nasibulin AG (2019). Dry-transfer technique for polymer-free single-walled carbon nanotube saturable absorber on a side polished fiber. Opt Mater Express.

[9] Sheng Q, Feng M, Xin W, Han T, Liu Y, Liu Z, Tian J (2013). Actively manipulation of operation states in passively pulsed fiber lasers by using graphene saturable absorber on microfiber. Opt Express.

[10] Lee H, Kwon WS, Kim JH, Kang D, Kim S (2015). Polarization insensitive graphene saturable absorbers using etched fiber for highly stable ultrafast fiber lasers. Opt Express.

[11] Chen H, Chen Y, Yin J, Zhang X, Guo T, Yan P (2016). High-damage-resistant tungsten disulfide saturable absorber mirror for passively q-switched fiber laser. Opt Express.

[12] Ma P, Lin W, Zhang H, Xu S, Yang Z (2019). High-power large-energy rectangular mode-locked Er-doped fiber laser based on high-damage-threshold mos2 saturable absorber. IEEE Photonics J.

[13] Han X, Zhang H, Jiang S, Zhang C, Li D, Guo Q, Gao J, Man B (2019). Improved laser damage threshold of In2Se3 saturable absorber by PVD for high-power mode-locked Er-doped fiber laser. Nanomaterials.

[14] Ostojic G, Zaric S, Kono J, Strano M, Moore V, Hauge R, Smalley R (2004). Interband recombination dynamics in resonantly excited single-walled carbon nanotubes. Phys Rev Lett.

[15] Martinez A, Sun Z (2013). Nanotube and graphene saturable absorbers for fibre lasers. Nat Photonics.

[16] Steinberg D, Rosa HG, de Souza EAT (2017). Influence of carbon nanotubes saturable absorbers diameter on mode-locking erbium-doped fiber laser performance. J Lightwave Technol.

[17] Schibli T, Minoshima K, Kataura H, Itoga E, Minami N, Kazaoui S, Miyashita K, Tokumoto M, Sakakibara Y (2005). Ultrashort pulse-generation by saturable absorber mirrors based on polymer-embedded carbon nanotubes. Opt Express.

[18] Shen B, Zhai W, Lu D, Wang J, Zheng W (2012). Ultrasonication-assisted direct functionalization of graphene with macromolecules. RSC Adv.

[19] Sun Y-P, Fu K, Lin Y, Huang W (2002). Functionalized carbon nanotubes: properties and applications. Acc Chem Res.

[20] Cheng Q, Debnath S, O’Neill L, Hedderman TG, Gregan E, Byrne HJ (2010). Systematic study of the dispersion of SWNTs in organic solvents. J Phys Chem C.

[21] Sahoo NG, Rana S, Cho JW, Li L, Chan SH (2010). Polymer nanocomposites based on functionalized carbon nanotubes. Prog Polym Sci.

[22] Liu C-X, Choi J-W (2012). Improved dispersion of carbon nanotubes in polymers at high concentrations. Nanomaterials.

[23] Soleimani H, Yahya N, Baig M, Khodapanah L, Sabet M, Burda M, Oechsner A, Awang M (2015). Synthesis of carbon nanotubes for oil-water interfacial tension reduction. Oil Gas Res.

[24] Atchudan R, Cha BG, Lone N, Kim J, Joo J (2019). Synthesis of high-quality carbon nanotubes by using monodisperse spherical mesoporous silica encapsulating iron oxide nanoparticles. Korean J Chem Eng.

[25] Futaba DN, Yamada T, Kobashi K, Yumura M, Hata K (2011). Macroscopic wall number analysis of single-walled, double-walled, and few-walled carbon nanotubes by X-ray diffraction. J Am Chem Soc.

[26] Aftab S, Hussain S, Siddique M, Nawaz H (2010). Comprehensive study of trends in the functionalization of CNTs using same oxidizing acids in different conditions. Der Pharm Chem.

[27] Qi G-Q, Cao J, Bao R-Y, Liu Z-Y, Yang W, Xie B-H, Yang M-B (2013). Tuning the structure of graphene oxide and the properties of poly (vinyl alcohol)/graphene oxide nanocomposites by ultrasonication. J Mater Chem A.

[28] Sankal S, Kaynak C (2013). Using various techniques to characterize oxidative functionalized and aminosilanized carbon nanotubes for polyamide matrix. J Reinf Plast Compos.

[29] Miyata Y, Yanagi K, Maniwa Y, Tanaka T, Kataura H (2008). Diameter analysis of rebundled single-wall carbon nanotubes using X-ray diffraction: verification of chirality assignment based on optical spectra. J Phys Chem C.

[30] Nakamiya T, Ueda T, Ikegami T, Ebihara K, Tsuda R (2008). Thermal analysis of carbon nanotube film irradiated by a pulsed laser. Curr Appl Phys.

[31] Costa S, Borowiak-Palen E, Kruszynska M, Bachmatiuk A, Kalenczuk R (2008). Characterization of carbon nanotubes by Raman spectroscopy. Mater Sci Pol.

[32] Yang D-Q, Rochette J-F, Sacher E (2005). Functionalization of multiwalled carbon nanotubes by mild aqueous sonication. J Phys Chem B.

[33] Girão EC, Liebold-Ribeiro Y, Batista JA, Barros EB, Fagan SB, Mendes Filho J, Dresselhaus MS, Souza Filho AG (2010). Functionalization of single-wall carbon nanotubes through chloroform adsorption: theory and experiment. Phys Chem Chem Phys.

[34] Stancu V, Galatanu A, Enculescu M, Onea M, Popescu B, Palade P, Aradoaie M, Ciobanu R, Pintilie L (2021). Influences of dispersions’ shapes and processing in magnetic field on thermal conductibility of PDMS-FE3O4 composites. Materials.

[35] Kim JH, Hwang J-Y, Hwang HR, Kim HS, Lee JH, Seo J-W, Shin US, Lee S-H (2018). Simple and cost-effective method of highly conductive and elastic carbon nanotube/polydimethylsiloxane composite for wearable electronics. Sci Rep.

[36] Shahzad M, Giorcelli M, Shahzad N, Guastella S, Castellino M, Jagdale P, Tagliaferro A (2013). Study of carbon nanotubes based polydimethylsiloxane composite films. J Phys Conf Ser.

[37] Silva EAd, Windmöller D, Silva GG, Figueiredo KCdS (2017). Polydimethylsiloxane membranes containing multi-walled carbon nanotubes for gas separation. Mater Res.

[38] Javanmardi MS, Ameri E (2020). Pervaporation characteristics of PDMS/PMHS nanocomposite membranes inclusive multi-walled carbon nanotubes for improvement of acetic acid-methanol esterification reaction. Polym Bull.

[39] Ouyang M, Yuan C, Muisener R, Boulares A, Koberstein J (2000). Conversion of some siloxane polymers to silicon oxide by UV/ozone photochemical processes. Chem Mater.

[40] Wu H, Xia H, Zhang X, Zhang H, Liu H, Sun J (2020). Polydimethylsiloxane/multi-walled carbon nanotube nanocomposite film prepared by ultrasonic-assisted forced impregnation with a superior photoacoustic conversion efficiency of 9.98 × 10^−4^. J Nanophoton.

[41] Shen X-J, Liu Y, Xiao H-M, Feng Q-P, Yu Z-Z, Fu S-Y (2012). The reinforcing effect of graphene nanosheets on the cryogenic mechanical properties of epoxy resins. Compos Sci Technol.

[42] Ryu SY, Kim K-S, Kim J, Kim S (2012). Degradation of optical properties of a film-type single-wall carbon nanotubes saturable absorber (SWNT-SA) with an Er-doped all-fiber laser. Opt Express.

[43] Hussain S, Shah KA, Islam S (2013). Investigation of effects produced by chemical functionalization in single-walled and multi-walled carbon nanotubes using raman spectroscopy. Mater Sci Pol.

[44] Jhon YI, Lee JH (2021). Saturable absorption dynamics of highly stacked 2d materials for ultrafast pulsed laser production. Appl Sci.

[45] Hernandez-Romano I, Davila-Rodriguez J, Mandridis D, Sanchez-Mondragon JJ, May-Arrioja DA, Delfyett PJ (2011). Hybrid mode locked fiber laser using a PDMS/SWCNT composite operating at 4 GHz. J Lightwave Technol.

