# Supplementary material for: Highly Damage-Resistant Thin Film Saturable Absorber Based on Mechanically Functionalized SWCNTs
Source: Nanoscale Res Lett. 2022 Jan 15;17:11. doi: 10.1186/s11671-021-03648-2 (PMC8761199; doi:10.1186/s11671-021-03648-2)
Supplement: Supplementary file 1 — Additional file 1. Figure S1. Change of (002) peak in XRD patterns according to ultrasonication time. Figure S2. Normalized saturable absorption of the SWCNT-PDMS SA. [file 11671_2021_3648_MOESM1_ESM.pdf]

Supplementary Information

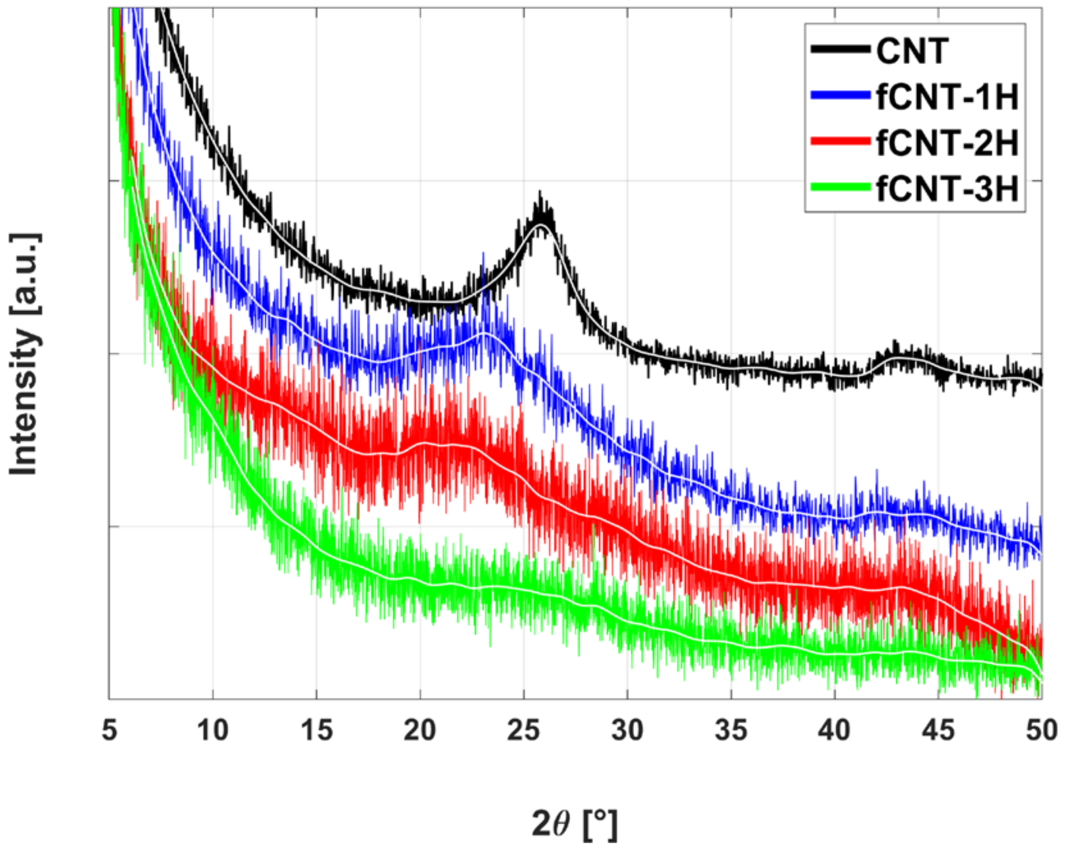

Figure S1 Change of (002) peak in XRD patterns according to ultrasonication time.

The white lines represent the fitting result through a Gaussian window for each XRD pattern.

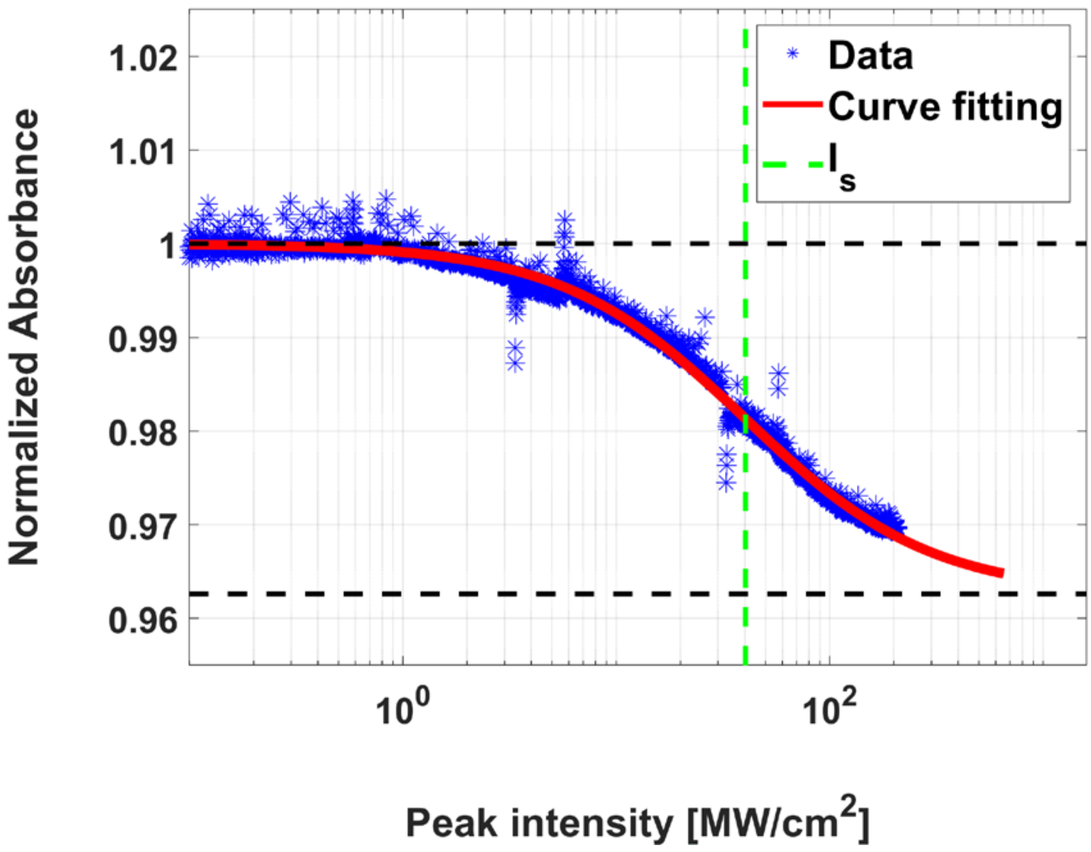

Figure S2 Normalized saturable absorption of the SWCNT-PDMS SA.
